# Supplementary material for: mRNA stem-loops can pause the ribosome by hindering A-site tRNA binding
Source: eLife. 2020 May 19;9:e55799. doi: 10.7554/eLife.55799 (PMC7282821; doi:10.7554/eLife.55799)
Supplement: Supplementary file 1. — The SD sequence is shown in green, slippery sequence in magenta, FSS in red, and handle sequence, which is complementary to biotinylated DNA oligo, underlined. To change the linker length between the HIV FSS and the P-site codon, HIV_NS12-nt linker mRNA and HIV_NS13-nt linker mRNA were prepared by extending the linker from native 11 nucleotides to 12 and 13 nucleotides, respectively. To alter codon identities, a UAC-to-GAG mutation was made on HIV_NS mRNA to generate HIV_NS (GAG) mRNA. Similarly, AUG-to-GAG and UUC-to-AUG mutations were made to generate HIV_NS (AUG) mRNA. The codon replacements are colored orange. Vertical black bars indicate the 3’ ends of ΔFSS mRNAs. [file elife-55799-supp1.docx]

Supplementary Materials

mRNA stem-loops can pause the ribosome by hindering A-site tRNA binding

Chen Bao, Sarah Loerch, Clarence Ling, Andrei A. Korostelev, Nikolaus Grigorieff and Dmitri N. Ermolenko

| **mRNA** | **Sequence** | **Restriction enzymes used for template linearization of FSS / ΔFSS mRNA variants** |
| --- | --- | --- |
| **dnaX _Slip** | 5’GGUUUUUCUUCUGAAGAUAAAGCAACAACAACAAGGCAAAGGGAGCAACCAUGGUAAAAAAGAGAGAA**CCGG\|CAGCCGCUACCCGCGCGCGGCCGG**UGAAUAACGGGAUC 3’ | **BamHI / ApeKI** |
| **dnaX_NS** | 5’GGUUUUUCUUCUGAAGAUAAAGCAACAACAACAAGGCAAAGGGAGCAACCAUGGUAUUCUACAGAGAA**CCGG\|CAGCCGCUACCCGCGCGCGGCCGG**UGAAUAACGGGAUC 3’ | **BamHI / ApeKI** |
| **HIV_NS** | 5’GGUUUUUCUUCUGAAGAUAAAGCAACAACAACAAGGCAAGGAGGUAAAAAUGUUCUACAA**\|**GAU**CUGGCCUUCCCACAAGGGAAGGCCAG**GGAA 3’ | **SacI / BglII** |
| **HIV_NS_12-nt linker_** | 5’GGUUUUUCUUCUGAAGAUAAAGCAACAACAACAAGGCAAGGAGGUAAAAAUGUUCUACAAGAAU**CUGGCCUUCCCACAAGGGAAGGCCAG**GGAA 3’ | **SacI /** |
| **HIV_NS_13-nt linker_** | 5’GGUUUUUCUUCUGAAGAUAAAGCAACAACAACAAGGCAAGGAGGUAAAAAUGUUCUACGGAAGAU**CUGGCCUUCCCACAAGGGAAGGCCAG**GGAA 3’ | **SacI /** |
| **HIV_NS (GAG)** | 5’GGUUUUUCUUCUGAAGAUAAAGCAACAACAACAAGGCAAGGAGGUAAAAAUGUUCGAGAA**\|**GAU**CUGGCCUUCCCACAAGGGAAGGCCAG**GGAA 3’ | **SacI / BglII** |
| **HIV_NS (AUG)** | 5’GGUUUUUCUUCUGAAGAUAAAGCAACAACAACAAGGCAAGGAGGUAAAAGAGAUGUACAA**\|**GAU**CUGGCCUUCCCACAAGGGAAGGCCAG**GGAA 3’ | **SacI / BglII** |

**Supplementary Table 1. Model mRNA sequences.** The SD sequence is shown in green, slippery sequence in magenta, FSS in red, and handle sequence, which is complementary to biotinylated DNA oligo, underlined. To change the linker length between the HIV FSS and the P-site codon, HIV_NS_12-nt linker_ mRNA and HIV_NS_13-nt linker_ mRNA were prepared by extending the linker from native 11 nucleotides to 12 and 13 nucleotides, respectively. To alter codon identities, a UAC-to-GAG mutation was made on HIV_NS mRNA to generate HIV_NS (GAG) mRNA. Similarly, AUG-to-GAG and UUC-to-AUG mutations were made to generate HIV_NS (AUG) mRNA. The codon replacements are colored orange. Vertical black bars indicate the 3’ ends of ΔFSS mRNAs.
